# Exocyst Sec5 Regulates Exocytosis of Newcomer Insulin Granules Underlying Biphasic Insulin Secretion

**DOI:** 10.1371/journal.pone.0067561

**Published:** 2013-07-02

**Authors:** Li Xie, Dan Zhu, Youhou Kang, Tao Liang, Yu He, Herbert Y. Gaisano

**Affiliations:** Department of Medicine, University of Toronto, Toronto, Ontario, Canada; University of Bremen, Germany

## Abstract

The exocyst complex subunit Sec5 is a downstream effector of RalA-GTPase which promotes RalA-exocyst interactions and exocyst assembly, serving to tether secretory granules to docking sites on the plasma membrane. We recently reported that RalA regulates biphasic insulin secretion in pancreatic islet β cells in part by tethering insulin secretory granules to Ca^2+^ channels to assist excitosome assembly. Here, we assessed β cell exocytosis by patch clamp membrane capacitance measurement and total internal reflection fluorescence microscopy to investigate the role of Sec5 in regulating insulin secretion. Sec5 is present in human and rodent islet β cells, localized to insulin granules. Sec5 protein depletion in rat INS-1 cells inhibited depolarization-induced release of primed insulin granules from both readily-releasable pool and mobilization from the reserve pool. This reduction in insulin exocytosis was attributed mainly to reduction in recruitment and exocytosis of newcomer insulin granules that undergo minimal docking time at the plasma membrane, but which encompassed a larger portion of biphasic glucose stimulated insulin secretion. Sec5 protein knockdown had little effect on predocked granules, unless vigorously stimulated by KCl depolarization. Taken together, newcomer insulin granules in β cells are more sensitive than predocked granules to Sec5 regulation.

## Introduction

The delivery of secretory granules to spatially restricted areas of the plasma membrane is a multistage process requiring polarized transport, restricted tethering, docking, and fusion of granules to specific regions on the plasma membrane. Each of these steps requires a discrete set of proteins to achieve high specificity. SNARE (soluble NSF attachment protein receptor) proteins mediate late-stage granule docking and subsequent fusion [1]. Tethering occurs after delivery of secretory granules from cell interior by cytockeletal motors to cell surface where a physical but reversible interaction between secretory granule and plasma membrane occurs, prior to interactions of SNARE proteins on opposing membranes [2]–[4]. The exocyst complex is an evolutionarily conserved multisubunit protein complex implicated in tethering secretory granules to the plasma membrane, which was originally identified in the budding yeast Saccharomyces cerevisiae, where the exocyst has been shown to be essential for exocytosis [2]–[5]. Loss of exocyst function still allowed secretory granule to be delivered to exocytic sites but could not undergo fusion [6], in part because SNARE complexes could not form [7]. The mammalian exocyst (called Sec6/8 complex), is an octameric protein complex (Sec3, Sec5, Sec6, Sec8, Sec10, Sec15, Exo70 and Exo84) that has also been implicated in tethering secretory vesicles to specific regions on the plasma membrane [8], [9]. Many small GTPase interact with the Sec6/8 complex, and GTP-dependent exocytosis was described in secretory β cells [10], [11] and pituitary gonadogrophs [12].

The exocyst components Sec5 and Exo84 were found to bind RalA and RalB in a GTP-dependent manner [13]–[17]. Here, the GTP-activation of Ral induces interaction with Sec5, which is required for assembly of exocyst complexes [18]. Recent studies showed that Ral GTPase can interact with Sec5 at the plasma membrane and Exo84 on the secretory vesicles [19], [20]. Release of the Ral-exocyst interactions may be triggered by phosphorylation events of Sec5, possibly leading to dissociation of the exocyst from vesicles or disassembly of the complex [21], [22]. Sec6/8 exocyst complex proteins were also shown to be present in pancreatic islet β cells, and dominant–negative mutants of Sec6 and Sec8 inhibited insulin secretion [23]. We [24] and others [25] showed that RalA regulates biphasic insulin secretion in pancreatic islet β cells by affecting different steps of the insulin secretory process. We subsequently showed that one of these steps is that RalA acts to tether insulin secretory granules to Ca^2+^ channels to assist excitosome assembly through binding Ca_v_ auxiliary subunit α_2_δ-1 [26].

In this work, we examined the role of Sec5 in insulin exocytosis. Sec5 is expressed in human and rodent islets, and INS-1 832/13 cells, and concentrated on insulin secretory granules. Sec5 protein knockdown (KD) in INS-1 832/13 cells inhibits patch clamp depolarization-induced exocytosis, attributed to a reduction of both the readily-releasable pool (RRP) and subsequent mobilization of the reserve pool. Employing total internal reflection fluorescence (TIRF) microscopy, we recently elucidated the Munc18/SNARE complex proteins that mediate exocytosis of newcomer insulin granules [27]–[30], which unlike predocked insulin granules, undergo little to no docking time at the plasma membrane. Newcomer insulin granules are a far larger contributor than predocked granules to biphasic glucose-stimulated insulin secretion (GSIS) [27]–[29]. Here, we further found that Sec5 protein KD had far greater effect in reducing recruitment and exocytosis of newcomer insulin granules than predocked granules, unless the latter was vigorously stimulated by KCl stimulation.

## Methods

### Cell Culture

Mice and rat islets were isolated by collagenase digestion method as previously described [31]. The islets were dispersed into single cells using a Ca^2+^/Mg^2+^-free phosphate-buffered saline (at 5 mM EDTA) with 0.25 mg/ml trypsin at 37°C for 5 minutes with gentle shaking and then resuspended in enriched RPMI-1640 media containing 11 mM D-glucose. The resulting cell suspensions were plated on glass coverslips and allowed to adhere approximately 48 hours before experiments. INS-1 832/13 cells (gift from Chris Newgard, Duke University, Durham, North Carolina) [32] were cultured in RPMI-1640 medium (GIBCO, Life Technologies, Burlington, Ontario, CA) with 10% FBS and penicillin/streptomycin at 37°C in an atmosphere of 5% CO_2_. All experimental procedures on rats and mice were approved by the Animal Care Committee of the University of Toronto.

### Western Blotting

Pancreatic islets were isolated from Wistar rat and Goto-Kakizaki (GK) rat (a gift from Claes-Goran Ostenson, Karolinska Institute, Stockholm, Sweden) [33], and Wistar rat brain, and INS-1 832-13 cells. Human islets from review board approved healthy donors (with either written informed consent from the donor or the next of kin obtained) were isolated using the Edmonton Protocol [34] and provided by the IsletCore of the University of Alberta, Canada; and its use approved by the Institutional Review Board at the University of Toronto. Lysate samples of these tissues were prepared and were separated on 12–15% gradient SDS-PAGE, transferred to nitrocellulose membrane and separated proteins identified by specific primary antibodies against Sec5 (1∶1,000; Rabbit anti-EXOC2; Proteintech Group, Chicago, IL, USA) and α-Tubulin (1∶10,000; mouse anti- α-Tubulin; SYSY, Goettingen, Germany).

### Confocal Immunofluorescence Microscopy

Immuno-staining was with primary antibodies: rabbit anti-Sec5 (Proteintech Group, Chicago, IL, USA) and guinea pig anti-insulin (DakoCytomation, Fort Collins, CO, USA); and secondary antibodies were anti-rabbit FITC, anti-guinea pig Texas Red and anti-rabbit Texas Red, respectively. Immunostained cells mounted on glass coverslips were examined using a laser scanning confocal imaging system (LSM510) equipped with the LSM software (Carl Zeiss, Oberkochen, Germany). Measurement of the fluorescent mean intensity was determined by NIS-Elements AR 3.0 (Nikon Inc., Japan). In our experiments, we chose the 63X objective and the 488, 543 laser to excite the fluorescein isothiocyanate (FITC) and Texas Red dyes, respectively.

### Lentivirus Transduction

Control (scrambled shRNA) and Sec5 shRNA lenti-viruses are gifts from Dr. David E. James (Garvan institute of medical research, Sydney, Australia). Lentiviruses were produced as previously described [24]. INS-1 832/13 cells were transduced with concentrated control (Htr-1) or Sec5 shRNA lentivirus, and stable cells lines were established by selection of GFP-positive cells using flow cytometry (BD Biosciences, Mississauga, ON, Canada). Cellular entry of viral particles after transduction was determined by GFP expression observed by epiflurescence imaging on Nikon TE2000U inverted microscope when performing the patch clamp and TIRF imaging studies.

### Electrophysiology

Patch electrodes were pulled from 1.5-mm thin-walled borosilicate glass, coated close to the tip with orthodontic wax (Butler, Guelph, Ontario, Canada), and polished to a tip resistance of 2–3 megaohms when filled with intracellular solution. For measurement of membrane capacitance, the intracellular solution contained: 125 mM Cesium glutamate, 10 mM CsCl, 10 mM NaCl, 1 mM MgCl_2_, 5 mM HEPES, 0.05 mM EGTA, 3 mM MgATP, and 0.1 mM cAMP (pH 7.2). The extracellular solution consisted of 118 mM NaCl, 5.6 mM KCl, 1.2 mM MgCl_2_, 10 mM CaCl_2_, 20 mM tetraethylammonium chloride, 5 mM HEPES, and 5 mM D-glucose (pH 7.4). Cell membrane capacitance (Cm) was estimated by the Lindau-Neher technique, implementing the “Sine-DC” feature of the Lock-in module (40 mV peak-to-peak and a frequency of 500 Hz) in the whole-cell configuration. Recordings were conducted using an EPC10 patch clamp amplifier and the Pulse and X-chart software programs (HEKA Electronik, Lambrecht, Germany). Exocytic events were elicited by a train of eight 500-ms depolarization pulses (1-Hz stimulation frequency) from -70 mV to 0 mV. All recordings were performed at 30°C.

### TIRFM and Data Analysis

Our TIRF microscope setup (Nikon, Toronto, Ontario, Canada) was constructed based on the prismless and through-the-lens configuration. Briefly, a condenser coupling multiple lasers (440 nm, 488 nm, 543 nm) was attached to the back port of our Nikon TE2000U inverted microscope, equipped with 60X oil immersion objective lens (NA = 1.49). We used a 488-nm beam to excite pHluorin, and 488RDC long-pass dichroic and 525/50-nm band-pass emission filters (Chroma). Images were collected with a cooled 16-bit EM-CCD camera (Cascade 512, Roper Scientific, USA). The penetration depth of the evanescent field (∼100 nm) was aligned by measuring the incidence angle of the 488-nm laser beam with a prism (n = 1.5163). Images were acquired at 5-Hz with a 100-ms exposure time. Insulin granules docking states were detected by Islet Amyloid Precursor Protein (IAPP)-mCherry (a gift from P. Rorsman, Oxford University, England), which is targeted to insulin-containing granules. Fusion events, indicated by abrupt brightening of syncollin-pHluorin fluorescence [35], were manually selected as we recently reported in detail [29]. Insulin granule mobilization and exocytosis were analyzed by Matlab (MathWorks, USA), ImageJ (NIH, USA), Igor Pro 5.03 (WaveMetrics, USA) softwares. A monolayer of INS-1 832/13 cells were infected with adenovirus encoding syncollin-pHluorin and further cultured in 36–48 hours before performing TIRFM. Before image acquisition, cells were pre-incubated for 30 mins in KRB buffer containing 2.8 mM glucose, and then were stimulated by KRB buffer containing 16.7 mM glucose or 50 mM KCl. All recordings were performed at 37°C.

### Statistical Analysis

Data are presented as the mean ± SEM with the indicated number of experiments. Statistical significance was evaluated by the Student’s t test or the Mann-Whitney rank sum test in SigmaStat 3.11 (Systat Software Inc. Chicago, IL, USA) and considered significant if P<0.05.

## Results

### Sec5 is Present in Islets and Localized to Insulin Granules

**Figure 1:**
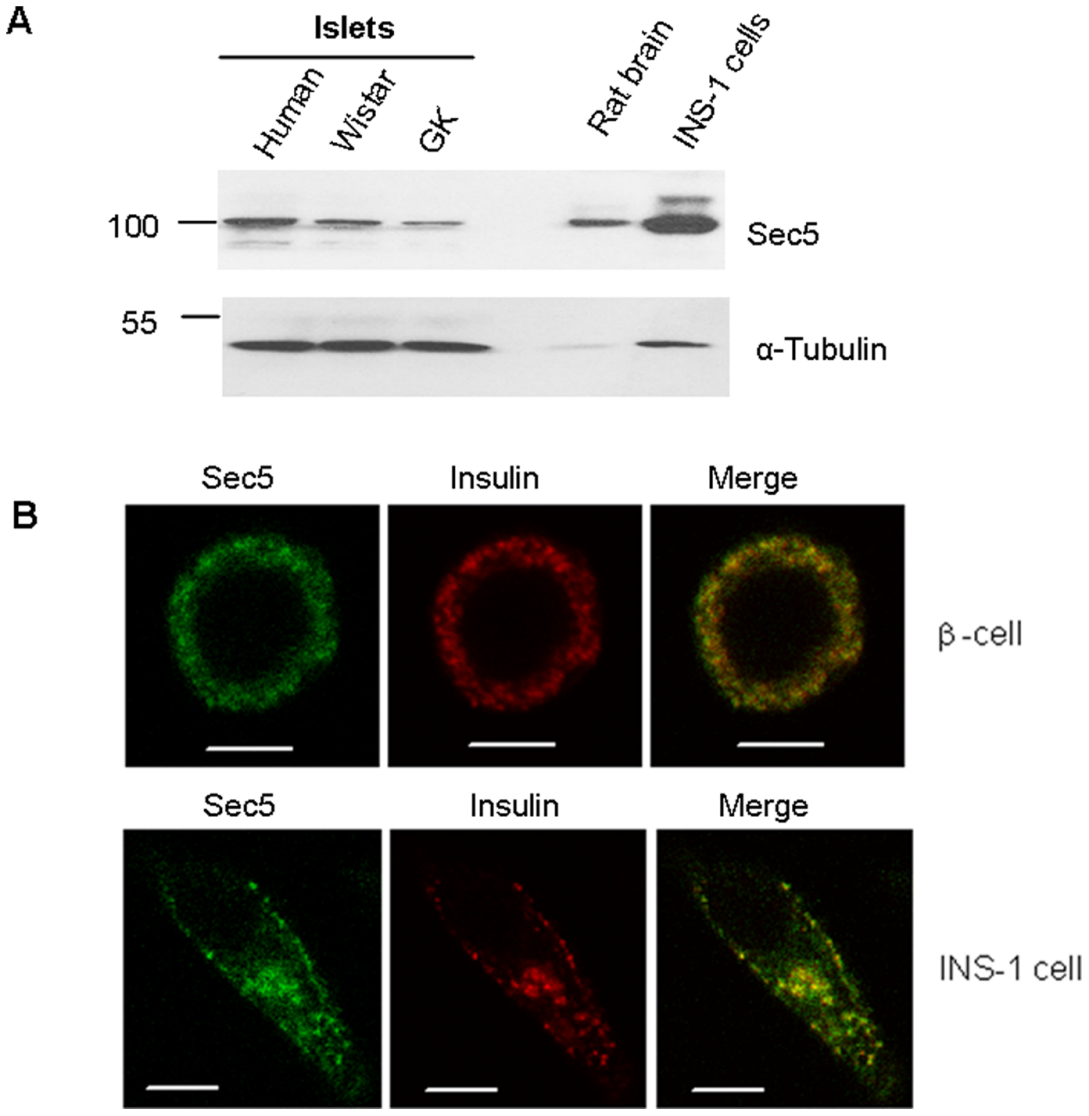
Sec5 is present in pancreatic islets and localized to insulin granules. (**A**) Western blotting shows Sec5 is present in human, Wister and GK rat islets, rat brain and INS-1 831/13 cells. α-tubulin indicates equal protein loading of pancreatic islets. (**B**) Confocal microscopy imaging shows Sec 5 is localized to insulin granules in a single mouse β cell and a single INS-1 832/13 cell. Bar, 5 µm.

Sec5 is present in human pancreatic islets and also in pancreatic islets of Wistar rats and the type 2 diabetic Wistar rat variant called Goto-Kakizaki (GK) rat, and the rat insulinoma cell line INS-1 832/13 (**Fig. 1A**). Islet Sec5 levels were however reduced in the GK rat, consistent with the reduced islet levels of SNARE proteins we previously reported in GK rats that in part explains the insulin secretory deficiency [33]. Confocal microscopy imaging revealed that Sec5 is localized to insulin granules in mouse pancreatic β cells and INS-1 832/13 cells (**Fig. 1B**).

### Sec5 KD Reduced Depolarization-induced Insulin Exocytosis

**Figure 2:**
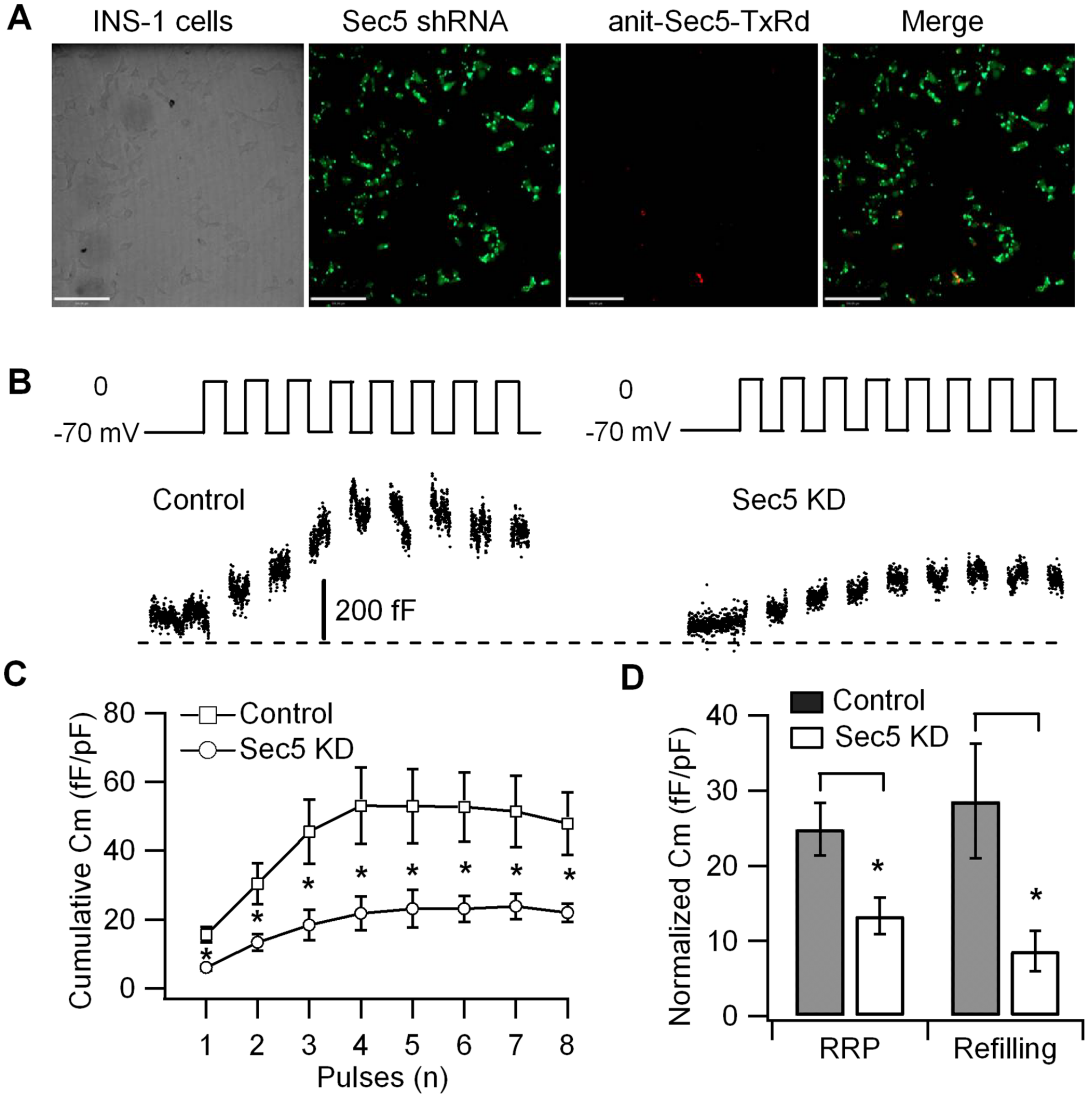
Sec5 regulates release of insulin granules from the readily-releasable and reserve pools. Changes in cell membrane capacitance (ΔCm) were measured from single INS-1 cells using a train of eight depolarizing pulses (500 ms in duration) from −70 mV to 0 mv. (**A**) Cofocal imaging shows that Sec5 protein expression in almost all INS-1 831/13 cells are knocked down by Sec5 lenti-shRNA, indicated by the GFP co-expression, and almost complete abrogation of Sec5-TxRd staining. (**B**) Representative recordings of capacitance from Htrl-1 (Control) lenti-shRNA and Sec5 lenti-shRNA (Sec5 KD) INS-1 832/13 cells. (**C**) Summary of the change in capacitance (ΔCm) normalized to basal cell membrane capacitance (fF/pF) from control (n  = 10 cells) and Sec5 KD (n  = 9) INS-1 832/13 cells. Values represent the mean ± SEM. *p<0.05. (**D**) Summary of the size of the RRP of insulin granules (ΔCm1^st^–2^nd^ pulse) and the rate of granule mobilization (ΔCm^3rd-^8^th^ pulse) (n  = 9–10 cells). Values represent the mean ± SEM. *p<0.05.

To examine the function of endogenous Sec5 on whole β cell insulin granule pool exocytotic kinetics, we employed the shRNA lentiviral technique to deplete endogenous Sec5 protein in the INS-1 832/13 insulinoma cell line. INS-1 832/13 very closely mimics the native islet β cell [32], and is excellent for both patch clamp membrane capacitance (Cm) recording of insulin granule exocytosis and TIRF microscopy imaging of exocytosis. However, INS-1 cells are notoriously difficult to transfect by conventional lipofection. We therefore employed viral infection, specifically infecting INS-1 832/13 cells with control or Sec5 shRNA-expressing lentiviruses, and stable cell lines were then created by selection of GFP-positive cells. Confocal imaging shows the INS-1 cells transduction efficiency of Sec5 shRNA lentivirus [tagged with green fluorescent protein (GFP)] was almost 100% (**Fig. 2A**). To confirm that the lentivirus shRNA-transduced cells (tagged with GFP) are depleted of Sec5, we performed immunostaining with anti-Sec5 antibody (secondary antibody is anti-rabbit Texas Red). In contrast to the abundance of Sec5 in untransfected INS-1 cells (**Fig. 1B** *lower panel*), GFP-expressing INS-1 cells were largely devoid of Sec5 staining, indicating that GFP-expressing cells are depleted of Sec5 (**Fig. 2A**).

We then performed patch clamp Cm measurements on the GFP-expressing cells infected with either scrambled shRNA (control) or Sec5 shRNA (Sec5 KD) INS-1 cells. Insulin exocytosis was elicited by a protocol consisting of a train of eight 500-ms depolarization pulses. Cell Cm changes elicited by the first two pulses would approximate the size of the RRP of primed and fusion-ready granules. Subsequent pulses would estimate the rate of granule refilling or mobilization from the reserve pool(s) to the RRP, where the granules are subsequently primed for fusion competence [36]. **Fig. 2B** shows representative recordings of capacitance from control and Sec5 KD INS-1 cells. When compared with control cells, the Cm increase in Sec5 KD was significantly inhibited at every depolarizing pulse (**Fig. 2C**). **Fig. 2D** shows that the size of the RRP of granules (ΔCm_1st-2ndpulse_) was significantly reduced by 46% in Sec5 KD cells (13.4±2.4 fF/pF) compared with control cells (24.9±3.5 fF/pF). In addition, the rate of granule refilling/mobilization (ΔCm_3rd-8thpulse_) was significantly reduced by 70% in Sec5 KD cells (8.7±2.7 fF/pF) compared with control cells (28.6±7.6 fF/pF). These results suggest that Sec5 knockdown greatly impairs depolarization-induced exocytosis by affecting release of the RRP of granules and mobilization of granules from reserve pool(s).

### Sec5 KD does not Affect Docking of the Population of Predocked Insulin Granules

**Figure 3:**
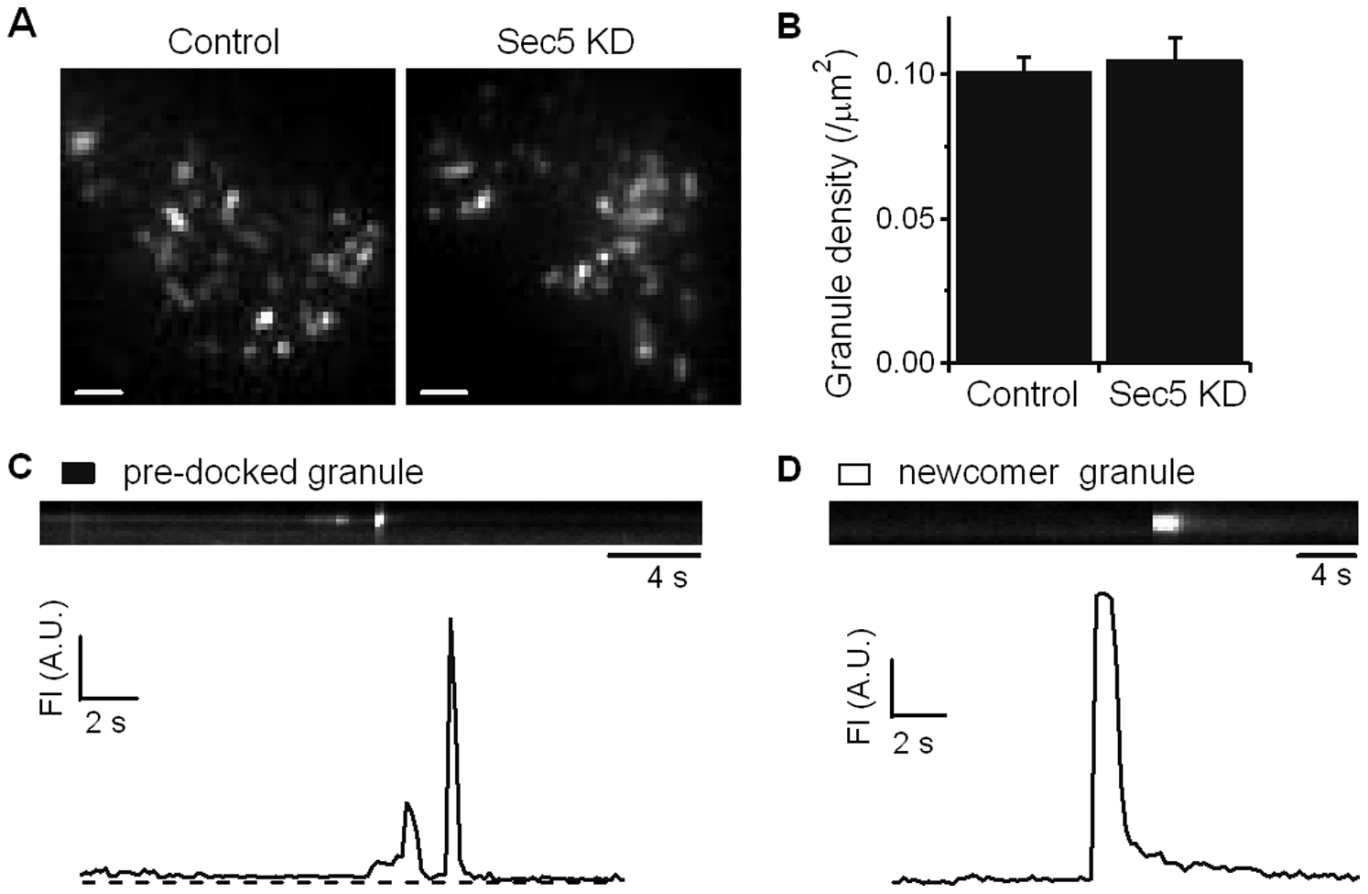
Sec5 depletion does not affect recruitment of insulin granules to dock onto the plasma membrane. (**A**) TIRF imaging of exocytosis of Control and Sec5 KD INS-1 832/13 cells infected with *Ad-*IAPP-mCherry. Scale bar 5 µm. (**B**) The graph shows a comparison of averaged granule densities from control and Sec5 KD INS-1 cells before stimulation (n = 10 cells for each). (**C–D**) Kymographs and the corresponding time-lapse fluorescence intensity (FI) curves indicate different fusion modes. (**C**) Fusion event of a pre-dock insulin granule; (**D**) fusion event of a newcomer granule that did not undergo a docking step on the plasma membrane before proceeding to exocytosis. A.U., arbitrary units.

TIRF microscopy was employed to assess single insulin granule exocytosis. We first examined the number of insulin secretory granules docked at the plasma membrane in unstimulated INS-1 cells. INS-1 cells were labeled with Adenovirus (Ad) Islet Amyloid Precursor Protein (IAPP)-mCherry in control and Sec5 KD cells. IAPP is a native cargo protein that is targeted to insulin granules, tagging with fluorophore has been used to visualize exocytosis of insulin granules in β cells [37]. Because the evanescent field generated by total internal reflection illuminates the fluorophores within ∼100-nm-thin layer underneath the plasma membrane, this allows capture with high spatial resolution the fluorescence behavior of IAPP-mCherry-tagged insulin granules. At basal unstimulated state (**Fig. 3A,B**), punctuate fluorescence indicating docked granules were not quantitatively different between control (0.101±0.005 per 100 µm^2^) and Sec5 KD INS-1 cells (0.105±0.008 per 100 µm^2^). This indicates that defects caused by Sec5 knockdown were not sufficient to disable insulin granules from docking onto the plasma membrane *per se* however, we could not decipher it there might be reduced docking at specific exocytotic sites on the plasma membrane.

To examine exocytosis *per se* by TIRF microscopy, we employed syncollin-pHluorin. Syncollin, a secretory granule protein originally found in pancreatic acinar cells, could be tagged with a pH-sensitive GFP variant, pHlourin, that fluoresces when exposed to the extracellular alkaline pH upon exocytosis of the secretory granule [35]; and which could be used as a surrogate granule cargo to tag insulin granules to assess exocytosis [38]. Control and Sec5 KD INS-1 cells were infected with adenovirus encoding syncollin-pHluorin and fusion events were indicated by abrupt brightening of pHluorin fluorescence followed by a cloud-like diffusion pattern indicating dispersion of the syncollin cargo into the cell exterior. Upon stimulation of the mouse β cells, we observed two distinct exocytotic fusion modes similar to our previous reports [27]–[29]. As shown in **Fig. 3C,D**, sequential images and corresponding time-lapse fluorescence intensity curves indicate the different fusion modes. ‘Pre-dock’ fusion mode (**Fig. 3C**) refers to granules already docked on the plasma membrane for a period of time before stimulation. ‘Newcomer’ fusion mode (**Fig. 3D**) refers to granules that are newly recruited and appear on the plasma membrane within the evanescent field after stimulation and then undergo exocytosis.

### Sec5 KD Causes Reduction in the Recruitment and Exocytosis of Newcomer Insulin Granules during Biphasic GSIS

**Figure 4:**
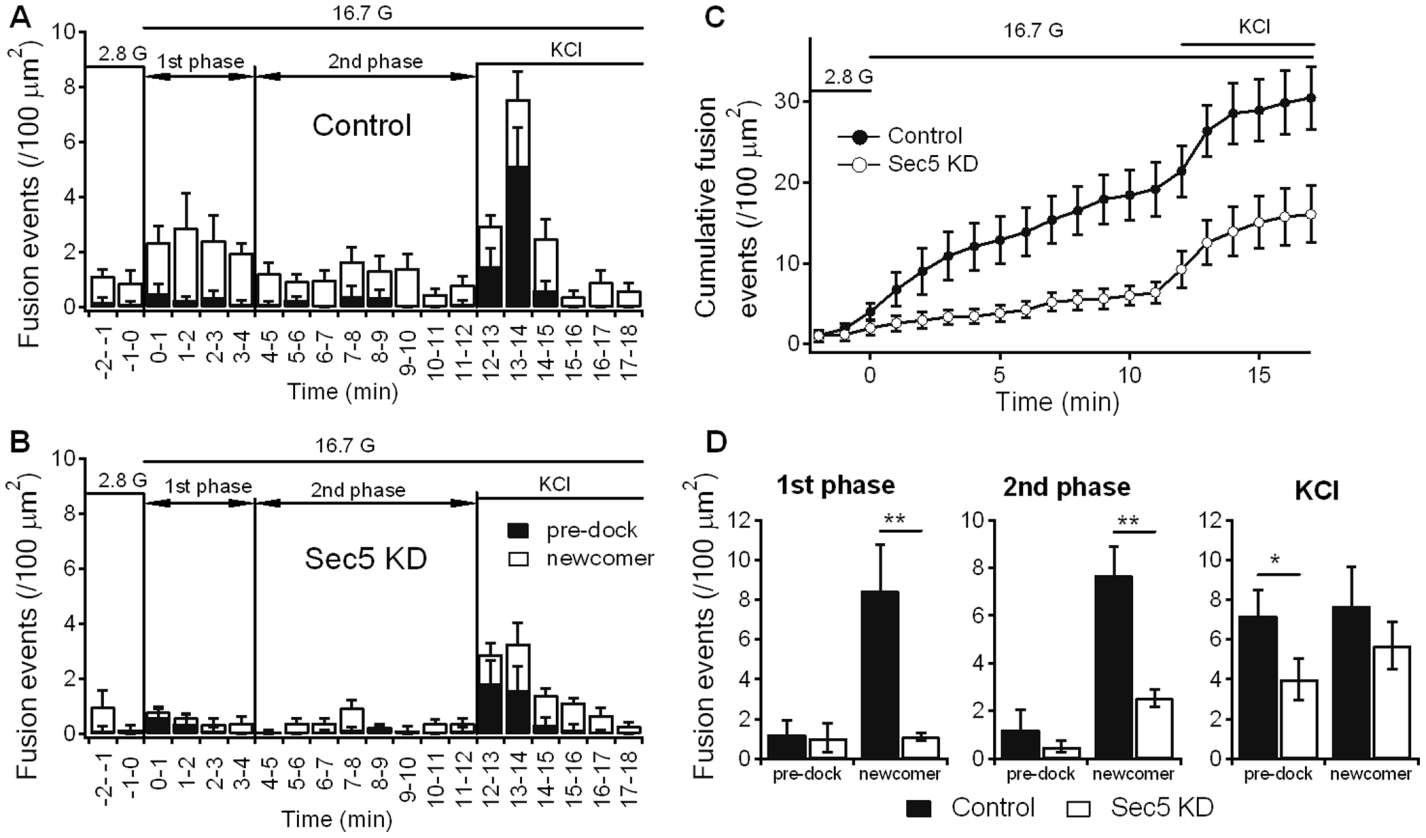
Sec5 depletion causes a reduction in the recruitment and exocytosis of newcomer insulin granules during biphasic GSIS. Control and Sec5 KD INS-1 832/13 cells were infected by *Ad*-syncollin-pHluorin. (**A, B**) Biphasic insulin granule exocytosis dynamics caused by 16.7 mM glucose (16.7 G) from Control (**A**) and Sec5 KD (**B**) INS-1 832/13 cells. Histograms of fusion events evoked by stimulation are as indicated in first phase (first 4 min after 16.7 mM glucose stimulation) and second phase (4–12 min after 16.7 mM glucose stimulation) GSIS; and subsequently after 50 mM KCl stimulation (13–18 min). *Black* and *open* bars indicate pre-docked granules and newcomer granules, respectively. Data were obtained from 10 cells for each condition, and expressed as mean ± SEM. (C) Normalized cumulative fusion events of insulin granules per unit area from Control (*black* circles) and Sec5 KD (*open* circles) INS-1 832/13 cells. (**D**) Comparison of sum of fusion events from pre-docked and newcomer granules in first phase and second phase after 16.7 mM glucose stimulation, and subsequent 50 mM KCl stimulation. Control (*black* bars); Sec5 KD (*open* bars). **p<0.01; *p<0.05.

At basal glucose (2.8 mM), we seldom found spontaneous fusion events (**Fig. 4A,B**). When stimulated with 16.7 mM glucose, single granule fusion events were then observed. In first-phase GSIS (first 4 minutes), newcomer granules (*open bars*) already accounted for >80% of exocytotic events in control INS-1 cells, and most of second phase GSIS (4–12 minutes, **Fig. 4A**). In Sec5 KD INS-1 cells, it is the reduction of newcomer granules (*open bars*, **Fig. 4B** **;** summary analysis in **Fig. 4D**) that accounted for the diverging cumulative increase in exocytosis in both phases of GSIS (*open circles*, **Fig. 4C**). In contrast, fusion events of predocked granules (*black bars*, **Fig. 4A,B**; summary analysis in **Fig. 4D**) were similar between control and Sec5 KD INS-1 cells in both first-phase and second-phase GSIS. Specifically, Sec5 KD caused a 86% reduction of newcomer granule release in first-phase GSIS (*open bars* in **Fig. 4A,B** and summary analysis in **Fig. 4D**; Sec5 KD: 1.11±0.2 per 100 um^2^ vs Control cells: 8.43±2.34 per 100 um^2^). In second-phase GSIS, newcomer granules release was reduced by 67% in Sec5 KD cells (Sec5 KD: 2.54±0.38 per 100 um^2^ vs Control cells: 7.68±1.23 per 100 um^2^). These results indicate that Sec5 depletion caused perturbation in the priming and fusion steps of newcomer granules during GSIS, but had little effect on the fusion of previously docked granules.

After the 16.7 mM glucose stimulation, the INS-1 cells were then treated with 50 mM KCl (12–18 minutes, **Fig. 4A,B** **and** analysis in **Fig. 4C,D**). KCl depolarization is known to preferentially trigger exocytosis of pre-docked granules [28]. Here, KCl indeed triggered exocytosis of previously docked granules and also some newcomer granules in both control and Sec5 KD cells (**Fig.** **4A,B**). However, only predocked granules release was significantly reduced by 44% in Sec5 KD cells (Sec5 KD: 4±1.06 per 100 um^2^ vs Control: 7.2±1.29 per 100 um^2^; **Fig. 4D**). This suggests that a more vigorous stimulation by KCl depolarization could uncover a fusion defect, albeit moderate defect in the predocked granules that was not previously observed with glucose stimulation. Peculiarly, KCl-stimulated newcomer granules did not seem to be significantly reduced by Sec5 KD probably because of the prior depletion of more releasable and primed newcomer granules in control cells during the initial glucose stimulation.

## Discussion

The pancreatic islet β cell is a unique secretory cell to test the exocytotic function of Sec5. Whereas much is known about the role of the exocyst complex in the plasma membrane tethering of predocked granules [2]–[5], how the exocyst affects newcomer granules is unknown.

In β cells, Sec5 KD surprisingly did not affect the number of predocked insulin granules on the β cell plasma membrane, suggesting that Sec5 may not affect the recruitment of insulin granules to the plasma membrane *per se*, which might be a function of cytoskeletal motors [39]. Rather, Sec5 KD might serve to reduce the docking of insulin granules at specific exocytotic sites on the plasma membrane. We had reported that RalA on insulin granules binds α_2_δ-1 auxiliary channel subunit to tether insulin granules to L- and R-type Ca^2+^ channels [26], postulated to mediate first and second phase GSIS, respectively [40]. It is possible that Sec5 on insulin granules would bind RalA to induce formation of exocyst complexes [18] that could assist in tethering insulin granules to these calcium channels. This would in part explain the exocytotic defect of predocked granules observed with KCl-stimulated depolarization that causes massive opening of these calcium channels. Perhaps a small residual amount of Sec5 may be sufficient given time to enable the defective exocyst complex to still partially perform its tethering function on the predocked granules. This milder defect on predocked granules was not manifested during glucose stimulation, as this is a mild stimulant on this granule pool. However, when β cells were vigorously stimulated by a membrane depolarizing stimuli, either electrically (patch clamp, on the RRP) or pharmacologically (KCl, on predocked granules), the fusion deficiency caused by Sec5 KD then became manifest, albeit still modest compared to the population of newcomer granules.

Newcomer granules undergo minimal to no docking time on the plasma membrane before undergoing exocytosis. Hence, it is intuitive that even a mild granule tethering defect caused by the Sec5 deficiency would have greater effect on the larger population of newcomer granules. In fact, our work showed that newcomer granules are far more vulnerable to the Sec5 deficiency, causing near complete abrogation of newcomer granule exocytosis during GSIS.

The various steps of exocytosis between predocked and newcomer insulin granules share both similar and distinct molecular machineries. In both, granule tethering to plasma membrane exocytotic sites involves Sec5 (this study) and RalA [24]–[26]. Granule priming in both predocked and newcomer granules require Munc13-1 [28], [31]. Nonetheless, the kinetics of insulin granule tethering and priming seem to occur at a more rapid pace for newcomer granules than for predocked granules. The Munc18 proteins mediating granule priming and SNARE complexes mediating granule exocytotic fusion *per se* are distinct, with predocked granules employing the same proteins as neurons (Munc18a, Syntaxin 1A, VAMP2), whereas newcomer granules employ Munc18b [30], Syntaxin 3 [29] and VAMP8 [27]. Inspite of much recent progress in this area, more work is still required to elucidate the molecular machinery underlying each exocytotic step of the newcomer granules. This is important since newcomer granules accounting for a much bigger portion of β-cell total insulin exocytotic capacity and could be targeted for more effective therapy to treat diabetes than the predocked granules [41].

In addition to exocytosis, Sec5 is also required for many aspects of membrane traffic within neurons [42], [43], and plays important role in facilitating protein transport to the apical rhabdomere in Drosophila photoreceptor cells [44]. Whether these Sec5 functions also apply to the islet β-cells will require further study.
